# Incidence of Ventilator-Associated Pneumonia in Critically Ill Children Undergoing Mechanical Ventilation in Pediatric Intensive Care Unit

**DOI:** 10.3390/children4070056

**Published:** 2017-07-03

**Authors:** Ali Amanati, Abdollah Karimi, Alireza Fahimzad, Ahmad Reza Shamshiri, Fatemeh Fallah, Alireza Mahdavi, Mahshid Talebian

**Affiliations:** 1 Pediatric Infections Research Center, Shahid Beheshti University of Medical Sciences, Tehran 1551415468, Iran; amanati@sums.ac.ir (A.A.); safahimzad@sbmu.ac.ir (A.F.); fafallah@sbmu.ac.ir (F.F.); 2 Department of Epidemiology and Biostatistics, School of Public Health, Tehran University of Medical Sciences, Tehran 1439955991, IR Iran; arshamshiri@tums.ac.ir; 3 Pediatric anesthesiologists and intensivist, Mofid children’s hospital, Shahid Beheshti Medical University, Tehran 1551415468, Iran; alirezamahdavi@sbmu.ac.ir; 4 BS of Nursery, Head Nurse of Pediatric Intensive Care Unit, Mofid children’s hospital, Shahid Beheshti University of Medical Sciences, Tehran 1551415468, IR Iran; mahshid.talebian69@gmail.com

**Keywords:** pneumonia, ventilator-associated, incidence, mortality, pediatric intensive care units

## Abstract

Background: Among hospital-acquired infections (HAIs) in children, ventilator-associated pneumonia (VAP) is the most common after blood stream infection (BSI). VAP can prolong length of ventilation and hospitalization, increase mortality rate, and directly change a patient’s outcome in Pediatric Intensive Care Units (PICU). Objectives: The research on VAP in children is limited, especially in Iran; therefore, the identification of VAP incidence and mortality rate will be important for both clinical and epidemiological implications. Materials and Methods: Mechanically ventilated pediatric patients were assessed for development of VAP during hospital course on the basis of clinical, laboratory and imaging criteria. We matched VAP group with control group for assessment of VAP related mortality in the critically ill ventilated children. Results: VAP developed in 22.9% of critically ill children undergoing mechanical ventilation. Early VAP and late VAP were found in 19.3% and 8.4% of VAP cases, respectively. Among the known VAP risk factors that were investigated, immunodeficiency was significantly greater in the VAP group (*p* = 0.014). No significant differences were found between the two groups regarding use of corticosteroids, antibiotics, PH (potential of hydrogen) modifying agents (such as ranitidine or pantoprazole), presence of nasogastric tube and total or partial parenteral nutrition administration. A substantial number of patients in the VAP group had more than four risk factors for development of VAP, compared to those without VAP (*p* = 0.087). Mortality rate was not statistically different between the VAP and control groups (*p* = 0.477). Conclusion: VAP is still one of the major causes of mortality in PICUs. It is found that altered immune status is a significant risk factor for acquiring VAP. Also, occurrence of VAP was high in the first week after admission in PICU.

## 1. Background

Given the high incidence of healthcare-associated infection, especially in resource-limited countries, infection-control practices and surveillance systems play an important role in improving patients’ safety, and decreasing the effect of life-threatening adverse events on healthcare systems. Healthcare-associated infections are usually underestimated in such countries [1]. Incidence of VAP ranged from 3% to more than 50% of ventilated PICU patients in different studies [2,3,4]. VAP incidence varies based on settings and geographical distribution [5]. Other important factors that may influence the reported rate include: study methodology [6,7], definition criteria (microbiological criteria versus non- microbiological criteria) [2,8], use of VAP prevention bundle programs [9], and medication practice in different PICUs [10].

Although there are some epidemiological studies that have been carried in neonatal intensive care units (NICU) in Iran [11], to the best of our knowledge, there is only one published study that has investigated the incidence of VAP in Iranian children in PICU [5,12].

The primary aim of this study is to describe the incidence of VAP, and the secondary aim is to determine the effect of VAP on mortality, and to determine risk factors for VAP in the Mofid Children’s Hospital PICU.

## 2. Patients and Methods

In this cross-sectional study, ventilated children were assessed regularly in the PICU of a tertiary teaching center in Tehran (the capital city of Iran) over 12 months in 2013–2014. The PICU throughout the study at Mofid Children’s Hospital was a 12-bed multidisciplinary care unit, with approximately 600 admissions annually. It is a mixed medical/surgical PICU wherein all patients are co-managed by pediatric pulmonologists and pediatric critical care physicians.

Any patient who needed respiratory support with mechanical ventilation was recruited to the study. The designed questionnaire was used to obtain demographic data and VAP assessment, which included risk factors and diagnostic criteria. Each patient was assessed by a single examiner (AA) within 24 hours of intubation (baseline), after 48 hours, and after 7 days of intubation (if still ventilated). Patients were evaluated for Centers for Disease Control and Prevention (CDC) criteria for VAP during the second and third assessments. [13,14,15].

Each patient with VAP fulfilled the imaging, laboratory and clinical criteria. Microbiological confirmation was not applied for diagnosis of ventilator-associated pneumonia based on CDC criteria [13]. Consecutive patients who met the VAP criteria were approached and recruited. We classified our cases as early-onset and late-onset VAP to determine the influence of timing of onset of pneumonia, which may have a possible effect on mortality attributable to VAP [16]. Among them, those who fulfilled VAP criteria during the first week of intubation (after 48 h of intubation) were considered to have early-onset VAP. Diagnosis of late-onset VAP was made for those who fulfilled VAP criteria after 7 days of intubation [17]. All patients were followed up until transfer to the ward for the mortality outcome. We matched the VAP group with a control group for assessment of VAP-related mortality in critically ill ventilated children.

Categorical data were reported as frequencies and percentages, and continuous data were reported as mean and standard deviation. The chi-square test was used to compare categorical data. This study was approved by the review board of the pediatric infections research center.

All procedures performed in studies involving human participants were in accordance with the ethical standards of the institutional and/or national research committee and with the 1964 Helsinki declaration and its later amendments or comparable ethical standards. This study was also approved by the Ethical Committee of the Pediatric Infections Research Center (PIRC) review board (number: 1392-1-91-11078-13443) in Shahid Beheshti University of Medical Science. Informed parental consent was obtained for all cases included in the study.

## 3. Results

Of 83 ventilated critically ill children in our PICU, 44 cases (53%) were male and 39 cases (47%) were female. The mean age of the patients was 29 months (the youngest was 1 month and the oldest was 12 years).

The incidence of VAP was 19/83 (22.9%) in mechanically ventilated patients. The three most common co-morbidities among patients with VAP were bacterial pneumonia, aspiration pneumonia and chronic heart failure (CHF), respectively. VAP developed in nearly all (94.7%) of the cases during the first week after admission to the PICU.

Early VAP was diagnosed in 16 patients (19.3%). Late VAP was diagnosed in 7 patients (8.4%). Among those with early VAP, late VAP was also detected in four cases. There was no significant difference regarding VAP-related mortality between those with and without VAP (*P* = 0.601). Furthermore, there was no significantly difference between early-onset and late-onset VAP in terms of VAP-related mortality (*P* = 0.533). Demographics, risk factors and mortality of children with and without VAP are summarized in Table 1.

Given the primary results of a simple logistic regression test, which was meaningful only for immune status, a multiple logistic regression test was not performed [18].

## 4. Discussion

In this cross-sectional descriptive study on mechanically ventilated PICU patients, a control group was considered to investigate the risk factors for acquisition of VAP and the associated clinical outcome at Mofid Children’s Hospital in Iran. The estimated incidence of VAP in our PICU was 22.9%, with a high mortality rate (47.4%). Among several VAP risk factors, only altered immune status was associated with higher risk of VAP. On the other hand, given the high-risk cases that were prone to VAP (more than about half of the cases), more intense VAP prevention strategies should have been considered in our PICU.

Incidence of VAP is differs greatly based on setting and location in critically ill children in PICU [5]. Overall, reported prevalence is about 3% to 27% [13,19]. Although VAP is considered as the second most common hospital-acquired infections in the PICU, after bloodstream infections (BSI) [13,14,20], reported incidence is higher in some studies [21,22]. El-Kholy et al. reported that VAP was the most commonly identified device-associated nosocomial infection (90%) among 490 pediatric patients [23]. Awasthi et al. revealed that VAP developed in 36.2% of children requiring mechanical ventilation in India [24]. Another recent national multicenter study on nosocomial infections in Spain, reported very low incidence of VAP (1.3%) among children undergoing mechanical ventilation in PICU [25]. Our estimated incidence is in line with another study conducted on Iranian children, reporting the incidence of VAP to be 27% [12].

Less is known about the risk factors of VAP in critically ill children in PICU. Previous studies reported that VAP mostly occurred in children who were ventilated for more than 4 days [24], or re-intubated [12]. Among investigated risk factors in our study, only altered immune status was statistically significant in those who developed VAP. It should be mentioned that the presence of the nasogastric tube, concurrent treatment with antibiotics, and PH-modifying agents such as ranitidine or pantoprazole were seen in nearly all children in the VAP and control group. Based on our results, the probability of a VAP event is greater during the first week of mechanical ventilation, which agrees with other reports [26,27]. Prolonged hospitalization prior to the onset of mechanical ventilation has been suggested by some researchers, and is probably an underappreciated risk factor for development of VAP, although we did not found it to be a significant risk factor [28].

We found that VAP has a high in-hospital mortality. The estimated mortality rate is about 5–14%, based on the limited available reports on VAP in children [5], but may be as high as 50%. VAP is considered to be an important risk factor for increasing mortality in infants and children in PICUs [14,29].

Our estimated mortality rate was 47.4%, which was extraordinarily high compared to many other reports [30,31]. High VAP-related mortality in our study could be attributed to younger age, co-morbidities, and lack of standard VAP Prevention Guidelines.

There are lots of reports that support applying VAP prevention programs to decrease the possibility of VAP among ventilated patients [32].

The small sample size may influence the statistical power of both risk factors and outcomes in this study. Also, the mortality in patients with no VAP was equally high (40.6%); hence, the high mortality cannot be attributed solely to VAP. It is likely that the overall mortality in our unit is high.

## 5. Conclusions

VAP is still one of the major causes of mortality in PICU. It was found that altered immune status is a major risk factor for acquiring VAP. Incidence of VAP was high in the first week after admission to the PICU. The results of this study emphasize the importance of applying early VAP prevention strategies in the PICU to reduce mortality. Further and larger prospective case-control studies are needed to evaluate the risk factors and outcomes of VAP.

## Figures and Tables

**Table 1:** Demographics, risk factors and mortality of children with and without ventilator-associated pneumonia (VAP) in the pediatric intensive care unit (PICU).

Variables (Total Number)	VAP n (%)	No VAP n (%)	*p*-Value
**Sex**			
Male/ Female (44/39)	10/9 (52.6/47.4)	34/30 (53.1/46.9)	0.970
**Age**			
<12 month (35)	10 (52.6)	25 (39.1)	0.390
1–5 years (33)	5 (26.3)	28 (43.8)	
>5 years (15)	4 (21.1)	11 (17.2)	
**Aspiration pneumonia**			
(n = 19)	3 (15.8)	16 (25.0)	0.158
**Corticosteroid usage**			
Yes/No (16/67)	5/14 (26.3/73.7)	11/53 (17.2/82.8)	0.376
**Partial Parenteral Nutrition**			
Yes/No (8/75)	3/16 (15.8/84.2)	5/59 (7.8/92.2)	0.301
**Nasogastric Tube**			
Yes/No (81/2)	19/0 (100/0)	62/2 (96.9/3.1)	0.435
**Antibiotic**			
Yes/No (83/0)	19/0 (100.0/0)	64/0 (100.0/0)	NA
**Immune status**			
Immunocompetent/ Immunocompromised (65/18)	11/8 (57.9/42.1)	54/10 (84.4/15.6)	0.014
**Intubation time from admission**			
Less than 48 h (82)	18 (94.7)	64 (100.0)	0.065
More than 48 h (1)	1 (5.3)	0 (0)	
**PH modifying agents**			
Yes/No (79/4)	19/0 (100.0/0)	60/4 (93.8/6.3)	0.264
Total number of positive investigated risk factors *			
Less than 4 (49)	8 (42.1)	41 (64.1)	0.087
More than 4 (34)	11 (57.9)	23 (35.9)	
**Survival**			
Survival/mortality (48/35)	10/9 (52.6/47.4)	38/26 (59.4/40.6)	0.601

* Includes: (1) corticosteroid therapy, (2) concurrent TPN administration during repeated sampling, (3) presence of nasogastric tube, (4) concurrent antibiotic treatment, (5) immunodeficiency disorders, (6) Intubation time from admission, and (7) concurrent PH (potential of hydrogen) modifying agents.
